# Diffuse myocardial fibrosis by T_1_-mapping in children with subclinical anthracycline cardiotoxicity: relationship to exercise capacity, cumulative dose and remodeling

**DOI:** 10.1186/1532-429X-15-48

**Published:** 2013-06-10

**Authors:** Edythe B Tham, Mark J Haykowsky, Kelvin Chow, Maria Spavor, Sachie Kaneko, Nee S Khoo, Joseph J Pagano, Andrew S Mackie, Richard B Thompson

**Affiliations:** 1 Department of Pediatrics, Stollery Children’s Hospital, University of Alberta, Edmonton, AB, Canada; 2 Faculty of Rehabilitation Medicine, University of Alberta, Edmonton, AB, Canada; 3 Department of Biomedical Engineering, University of Alberta, Edmonton, AB, Canada; 4 Division of Cardiology, Stollery Children’s Hospital, 4C2 Walter C Mackenzie Centre, 8440-112 Street, Edmonton, AB T6G 2B7, Canada

**Keywords:** CT, CMR, T1 mapping, Echocardiography, Contractile function, Cardiovascular imaging agents/Techniques, Exercise testing, Cardiac function, Cardiovascular magnetic resonance, Myocardial fibrosis, Speckle tracking echocardiography, T1 mapping

## Abstract

**Background:**

The late cardiotoxic effects of anthracycline chemotherapy influence morbidity and mortality in the growing population of childhood cancer survivors. Even with lower anthracycline doses, evidence of adverse cardiac remodeling and reduced exercise capacity exist. We aim to examine the relationship between cardiac structure, function and cardiovascular magnetic resonance (CMR) tissue characteristics with chemotherapy dose and exercise capacity in childhood cancer survivors.

**Methods:**

Thirty patients (15 ± 3 years), at least 2 years following anthracycline treatment, underwent CMR, echocardiography, and cardiopulmonary exercise testing (peak VO_2_). CMR measured ventricular function, mass, T_1_ and T_2_ values, and myocardial extracellular volume fraction, ECV, a measure of diffuse fibrosis based on changes in myocardial T_1_ values pre- and post-gadolinium. Cardiac function was also assessed with conventional and speckle tracking echocardiography.

**Results:**

Patients had normal LVEF (59 ± 7%) but peak VO_2_ was 17% lower than age-predicted normal values and were correlated with anthracycline dose (r = −0.49). Increased ECV correlated with decreased mass/volume ratio (r = −0.64), decreased LV wall thickness/height ratio (r = −0.72), lower peak VO_2_(r = −0.52), and higher cumulative dose (r = 0.40). Echocardiographic measures of systolic and diastolic function were reduced compared to normal values (p < 0.01), but had no relation to ECV, peak VO_2_ or cumulative dose.

**Conclusions:**

Myocardial T_1_ and ECV were found to be early tissue markers of ventricular remodeling that may represent diffuse fibrosis in children with normal ejection fraction post anthracycline therapy, and are related to cumulative dose, exercise capacity and myocardial wall thinning.

## Background

Despite the effectiveness of anthracycline chemotherapy, cardiotoxicity remains a significant long-term secondary effect. The myofibrillar loss and cellular necrosis from anthracycline cardiotoxicity leads to delayed and irreversible myocardial damage, culminating in cardiac dysfunction, cardiomyopathy and heart failure [1-3]. Current protocols reduce the maximum cumulative anthracycline dose to <400 mg/m^2^, but despite lower doses, patients are still at risk of ‘subclinical’ myocardial damage, i.e. prior to the development of clinical signs and symptoms [4]. The severity of cardiac dysfunction increases with the length of follow up, placing children at increased risk of myocardial dysfunction, and making the need for long-term monitoring in children more important [4]. Because of marked individual variation in cardiotoxicity, there is a need for a reliable and non-invasive method of early detection and serial monitoring for cardiotoxicity [2]. This may then guide early management and medical therapy in attempt to delay the onset of clinical cardiotoxicity.

Current oncology guidelines for the follow up of anthracycline chemotherapy are based on global indices of LV systolic function such as fractional shortening or ejection fraction (EF) [5]. Modern functional parameters such as speckle tracking echocardiography (STE) to measure strain and strain rate (SR) have been shown to be more sensitive than conventional functional parameters to detect early ventricular dysfunction in patients after cardiotoxic chemotherapy [6-9]. However, functional metrics alone provide limited insight into the underlying myocardial damage. Cardiovascular magnetic resonance (CMR) has the ability to characterize myocardial tissue using T_1_ and T_2_ mapping techniques. Quantitative T_1_ imaging, in particular, can be used to calculate the myocardial extracellular volume fraction (ECV), a measure of microscopic myocardial remodeling that has been associated with underlying diffuse fibrosis [10]. The aim of this study was to determine the CMR tissue characteristics (myocardial T_1_, T_2_ and derived ECV) in subclinical myocardial toxicity and their association with LV function and structure, exercise capacity and chemotherapy dose in childhood and adolescent cancer survivors.

## Methods

### Study design

We performed a cross-sectional study of eligible patients between 7 and 19 years, in remission for at least 2 years, who previously received anthracycline therapy, recruited from the Stollery Children’s Hospital Pediatric Oncology Survivor Program. Patients with a previous history of acute anthracycline cardiotoxicity or ventricular dysfunction were not excluded from the study. Subjects were excluded for known congenital heart disease, usual contraindications for CMR, inability to perform exercise testing, or living >2 hours outside the local area. Enrolled patients underwent CMR, echocardiography, and exercise testing on the same day. Written informed consent and assent were obtained from the parent and patient, and the study was approved by the University of Alberta Health Research Ethics Board. As all our patients had normal renal function, the use of gadolinium contrast agent was considered of low risk and clinically useful in terms of the ability to detect myocardial fibrosis using late enhancement techniques.

### CMR

CMR was performed on a 1.5 T Siemens Sonata scanner (Siemens Medical Solutions, Erlangen, Germany) using a 5-element (2 anterior, 3 posterior) cardiac array coil with end-expiratory breath-holds. Standard balanced steady-state free precession (SSFP) short-axis cines covering the left ventricle (LV) were obtained (echo time, TE 1.24 ms, repetition time, TR 2.89 ms, flip angle 51°, 8 mm slice thickness, 2 mm gap, matrix size 256 × 126, field of view 360 × 238 mm and 30 reconstructed cardiac phases). Quantitative T_2_ imaging was performed in a mid-ventricular short axis slice using a black-blood single-shot fast spin-echo pulse sequence with images acquired every 5^th^ heart beat at 3 different echo times (TE 23, 54 and 84 ms, slice thickness 8 mm, acquisition matrix size 192 × 108 and field of view 360 × 270 mm) [11].

**Figure 1:**
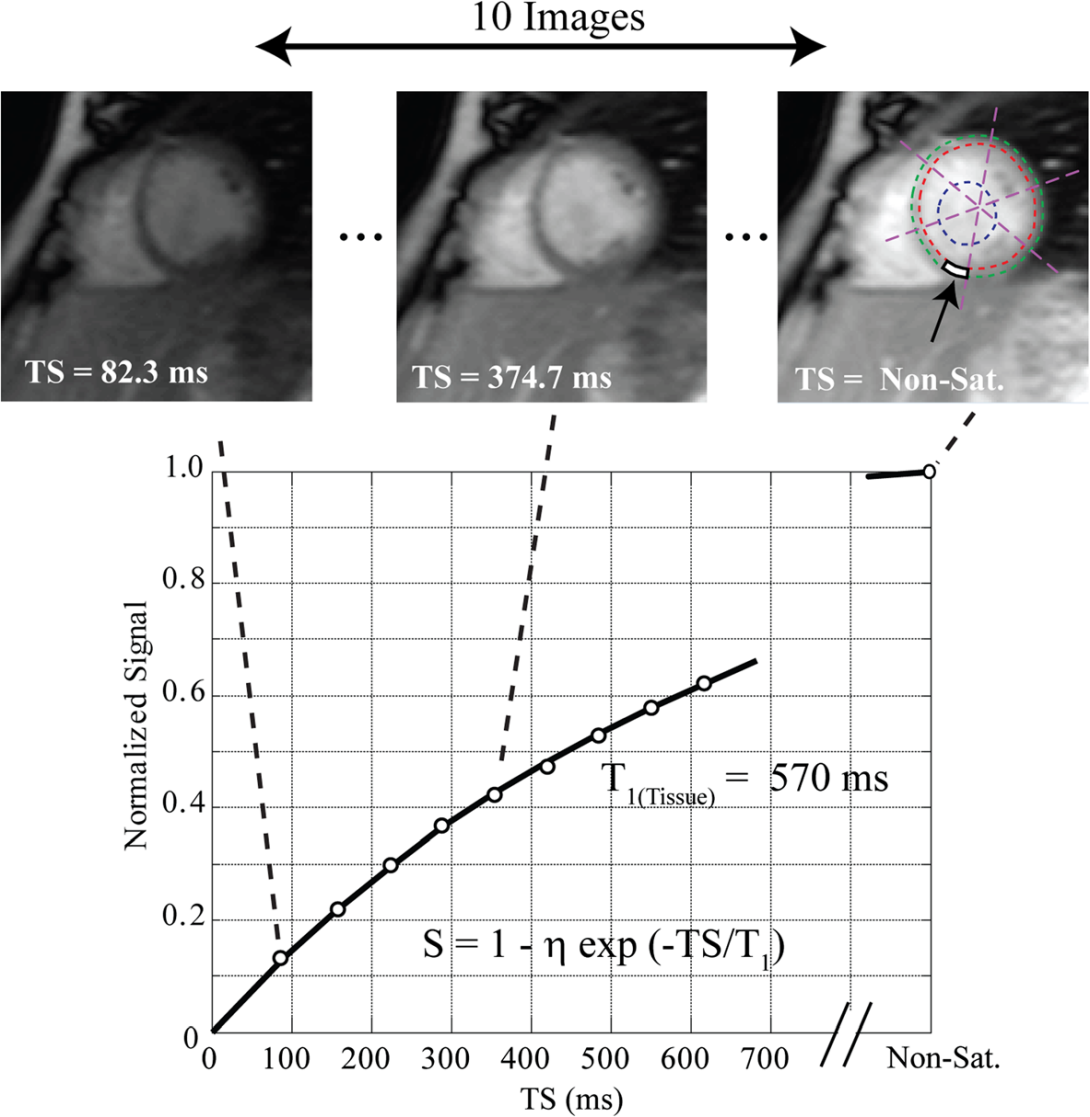
**SASHA T**_**1 **_**mapping method in a subject 15 minutes post-contrast.** On each of 10 saturation recovery images, the endocardium, epicardium and a region of interest in the blood are selected. The myocardium is automatically divided into 18 circumferential segments and T_1_ values are calculated in each segment by fitting a recovery curve as described in the methods. Average signal intensity in a septal segment (white box, far right image) are shown along with a best-fit saturation recovery curve. S is the measured signal intensity normalized to the non-saturated image intensity, η is the best-fit saturation efficiency, TS are saturation recovery times and T_1_ is the best fit T_1_ value.

Myocardial T_1_ mapping was performed in a matched mid-ventricular short axis slice using a custom SAturation-recovery single-SHot Acquisition (SASHA) SSFP pulse sequence, which has previously been validated with numerical simulations and phantoms, showing excellent agreement with gold standard spin-echo imaging, with no systematic errors [12,13]. For SASHA acquisitions, single-shot images were acquired during diastasis in sequential heartbeats, with a single non-saturated image followed by 9 images with saturation recovery times (TS) spanning the interval from the QRS to diastasis (Figure 1). Typical pulse sequence parameters: TE 1.39 ms, TR 2.78 ms, flip angle 70°, 8 mm slice thickness, matrix size 192 × 96, field of view 320 × 200 mm, 75% phase partial Fourier, and TS 82–720 ms for a heart rate of 60 bpm. T_1_ maps were acquired at baseline and 15 minutes after a bolus injection of 0.125 mmol/kg gadopentetatedimeglumine (Magnevist, Bayer Inc., Toronto, Canada). Conventional late gadolinium enhancement imaging was performed 7 minutes after contrast injection using a phase sensitive inversion recovery (PSIR) sequence in the short-axis, 2-, 3-, and 4-chamber views.

### CMR image processing

Quantification of left ventricular end-systolic (LVESV) and end-diastolic (LVEDV) volumes and mass were performed using cmr^42^ (Circle Cardiovascular Imaging, Calgary, Canada) to calculate LV ejection fraction, LVEF = (LVEDV-LVESV)/LVEDV. Papillary muscles were included in the left ventricular cavity. Mass and volumes were indexed to body surface area, while average wall thickness in diastole was indexed to height.

Epi- and endocardial contours were traced for T_1_ and T_2_ analysis using custom software (MATLAB, The MathWorks, Natick, MA, United States). The myocardial region was automatically segmented into 18 circumferential segments, referenced to the inferior right ventricular insertion point, in each of which the signal was averaged prior to fitting of signal intensity. For T_2_ analysis, signal intensity was fit using a Nelder-Mead simplex direct search algorithm to a mono-exponential decay, S = k*exp(−TE/T_2_), (S: signal intensity, k: a constant, TE: echo-time, and T_2:_ spin-spin relaxation time).

For T_1_ analysis, signal intensity was fit using a two-stage Nelder-Mead simplex direct search algorithm to a three-parameter mono-exponential recovery curve, S = k*(1-η*exp(−TS/T_1_)), (S: signal intensity, k: a constant, η: saturation efficiency, TS: saturation recovery times and T_1_: spin–lattice relaxation time, Figure 1). A region of interest drawn in the LV cavity was used to measure the blood T_1_. In each of the 18 segments, the extracellular volume fraction (ECV), which is the volume in which gadolinium contrast agent is distributed, was estimated using the calculated concentrations of contrast agent in the blood and tissue [10]. The contrast agent concentrations are proportional to the difference in 1/T_1_ values from baseline to after contrast delivery, resulting in the standard expression: ECV = [1/T_1_(myocardium_post_) – 1/T_1_(myocardium_pre_)]/[1/T_1_(blood_post_) – 1/T_1_(blood_pre_)] * (1-Hct), where Hct was the most recent blood hematocrit [10]. The contrast agent was assumed to be in equilibrium between the vascular and interstitial space by 15 minutes after injection, at the time of post-contrast T_1_ measurement [14]. All results for T_2_, T_1_ and derived ECV, are the average values from the 18 segments.

### Echocardiography

Two-dimensional echocardiography was performed using a Vivid 7 ultrasound platform (GE Medical Systems, Milwaukee, WI, United States). Traditional systolic and diastolic functional parameters measured included: fractional shortening (FS), peak early filling (E velocity), late filling (A velocity), medial and lateral LV tissue Doppler (TDI) peak systolic velocity (S’), peak early diastolic velocity (E’), peak late diastolic velocity (A’), and E/E’ ratios. The basal short axis and apical 4-chamber views were used to obtain speckle tracking echocardiography global circumferential and longitudinal strain, and global circumferential and longitudinal strain rate (SR) in systole and early diastole, during a breath-hold at end-expiration. Echocardiographic measures were analysed offline using EchoPAC software (version 7.1, GE Medical systems, Milwaukee, Wisconsin). The echocardiographic parameters were compared to 30 healthy normal controls of similar ages from our existing institutional database, who had previously undergone full functional echocardiography studies at rest using M-mode, 2D, TDI and STE measuring the same parameters.

### Cardiopulmonary exercise testing

Incremental exercise testing with expired gas analysis was performed on a cycle ergometer with an initial power output of 10–15 watts, increasing by 5–10 watts every 2 minutes according to the subject’s fitness capacity. Heart rate was continuously monitored with 12-lead ECG, and blood pressure measured every two-minutes with a cuff sphygmomanometer. Expired gases were collected with a commercially available metabolic measurement system (Parvomedics, Salt Lake City, UT, United States). Maximum oxygen consumption, peak VO_2,_ was determined as the highest oxygen consumed over a 30-second period.

### Statistical analysis

A Pearson correlation (|r|) greater than 0.48 would be detectable with 83% power assuming a 0.05 significance level (α) and null hypothesis correlation of 0, with 30 patients. Thirty patients also achieved an effect size of 0.46 to compare CMR parameters to known reported normal values with 80% power if α = 0.05. Assuming a standard deviation of 10, a 4.6 unit minimum decrease compared to normal MRI values would be detected. Power calculations were completed using PASS 2008.

Data are presented as mean ± standard deviation. Peak VO_2_ was compared with healthy reported age and gender matched normals [15]. Differences between patients who participated in the study and those who declined, were analysed using unpaired t-tests and Fisher’s exact tests. Differences between echocardiographic parameters of patients and controls, and gender differences in the novel MRI parameters were analysed using unpaired t-tests. Pearson’s product moment coefficient and linear regression were used to compare non-contrast myocardial T_1_ and ECV to continuous MRI values, anthracycline dose and peak VO_2_. Gender was included as an independent variable for all comparisons. A significance level of 0.05 was used. Statistical analysis was performed using SPSS (version 19, IBM Software Group, Somers, United States) and S-Plus 8.0 for Windows, Insightful Corp. 2008 (Vienna, Austria, 2011, version 2.12.2).

## Results

### Clinical characteristics

Out of 50 eligible patients, 30 agreed to participate with the following cancer diagnoses: 10 acute lymphocytic leukemia, 11 lymphomas (large cell, Burkitts, Hodgkins), 2 acute myelocytic leukemia, 2 Wilms’ tumour, 1 Ewing’s sarcoma, 2 neuroblastoma, 1 osteosarcoma, and 1 hemangiopericytoma. The major reasons for declining to participate were the need for intravenous access for MRI contrast administration and unwillingness to have an MRI scan. The characteristics of patients who participated did not differ from those who declined with regard to age, gender, anthracycline dose, and previous chest irradiation as shown in Table 1. There were 3 patients who had previous acute cardiotoxicity as evidenced by decreased LV function on echocardiography during the acute stage, with all 3 showing normal LV function at the time of the study. During the acute stage, 4 patients had septic events, 5 received radiation therapy, 5 received steroid treatment, and 7 required transfusions. Patients were not routinely screened for evidence of iron overload. Three patients received bone marrow transplantation. No patient had cardiovascular related symptoms or cardiovascular risk factors such as hypertension, diabetes, or obesity. None of the patients received cardioprotective agents.

**Table 1:** Characteristics of eligible subjects

	**Participated (n = 30)**	**Declined (n = 20)**	**p value**
Age (years)	15.2 ± 2.7	14.7 ± 2.4	0.17
Male (%)	50	70	0.72
Anthracycline dose (mg/m^2^)	197.2 ± 84.3	200.2 ± 83.0	0.92
Time from chemo (years)	7.6 ± 4.5	8.3 ± 5.0	0.90
Chest irradiation (%)	17%	25%	0.72

### Echocardiography parameters

Patients and controls used for comparison of echocardiography parameters were similar in age (15.2 ± 2.7 years versus 13.8 ± 3.4 years), gender (50% versus 57% male) and BSA (1.57 ± 0.3 versus 1.47 ± 0.3 m^2^). For conventional echocardiography parameters (Table 2), FS was significantly lower in patients compared with controls (p < 0.01), however it remained within the normal range. Similarly for several of the TDI parameters, there was a significant difference between patients and controls but with all values within the range of normal values. Patients also showed significantly decreased circumferential and longitudinal strain, longitudinal SR, and circumferential and longitudinal diastolic SR (p < 0.01) compared to controls (Table 3). However, these results were within the range of normal values reported in children [16]. There was no difference in circumferential SR between patients and controls. There was no significant relationship between any echocardiographic parameters and peak VO_2_ or anthracycline dose.

**Table 2:** Traditional 2D Echocardiography parameters of patients and controls

**Variables**	**Patients (n = 30)**	**Controls (n = 30)**	**p value**
FS (%)	34 ± 3	37 ± 4	<0.01
E velocity (cm/s)	82.3 ± 11.2	83.5 ± 19.9	NS
A velocity (cm/s)	43.0 ± 14	40.3 ± 11.3	NS
Lateral E’ velocity (cm/s)	12.6 ± 2.2	14.6 ± 2.1	0.001
Lateral A’ velocity (cm/s)	3.1 ± 1.2	4.2 ± 1.4	<0.01
Lateral S’ velocity (cm/s)	7.2 ± 2.3	7.7 ± 2.1	NS
Lateral E/E’	6.7 ± 1.4	5.9 ± 1.0	0.03
Medial E’ velocity (cm/s)	10.3 ± 1.5	11.0 ± 5.1	NS
Medial A’ velocity (cm/s)	3.4 ± 1.5	4.2 ± 2.1	0.05
Medial S’ velocity (cm/s)	6.0 ± 1.1	6.6 ± 0.8	0.01
Medial E/E’	8.1 ± 1.4	7.4 ± 1.5	0.03
Medial IVRT (ms)	59.9 ± 16.9	63.3 ± 11.9	NS

Abbreviations: *FS*: fractional shortening, *IVRT*: isovolumic relaxation time.

**Table 3:** Speckle tracking strain parameters of patients and controls

	**Patients (n = 30)**	**Controls (n = 30)**	**p value**
**Longitudinal**			
strain (%)	−18.1 ± 3.2	−21.0 ± 1.6	<0.01
SR (%/s)	−1.05 ± 0.2	−1.2 ± 0.12	<0.01
diastolic SR (%/s)	1.65 ± 0.5	1.99 ± 0.47	<0.01
**Circumferential**			
strain (%)	−17.8 ± 3.4	−19.9 ± 2.5	<0.01
SR (%/s)	−1.19 ± 0.22	−1.24 ± 0.18	NS
diastolic SR (%/s)	1.68 ± 0.49	2.06 ± 0.42	<0.01

Abbreviation: *SR*: strain rate.

### CMR

Left ventricular EDV and EF in patients were within normal reference ranges, while LVESV was slightly larger and LV mass slightly decreased compared to normal reference ranges reported in this age group (Table 4) [17,18]. There was no significant relationship between MRI-derived volumetric or functional parameters with either exercise capacity or anthracycline dose. All patients were negative for late gadolinium enhancement.

**Table 4:** CMR parameters of subjects

**CMR parameter**	**Result (mean + SD)**	**Range**
LVEF (%)	57.6 ± 4.9	41 - 74
LVEDVi (ml/m^2^)	87.8 ± 16.0	55.5 - 114.5
LVESVi (ml/m^2^)	37.4 ± 8.8	23.0 - 71.3
LV mass index (g/m^2^)	52.6 ± 12.6	33.7 - 99.2
Mass/volume ratio (g/ml)	0.6 ± 0.1	0.55 - 0.96
Wall thickness/height ratio	0.035 ± 0.005	0.027 – 0.046
Myocardial T_2_ (ms)	54.6 ± 4.9	43.2 - 65.7
Myocardial T_1_ (baseline, ms)	1155.3 ± 56.5	1074 - 1300
Blood T_1_ (baseline, ms)	1449.0 ± 215.2	1279-1839
Myocardial T_1_ (post-Gd, ms)	652.1 ± 53.6	540 - 750
Blood T_1_ (post-Gd, ms)	390.5 ± 51.6	310 - 498
ECV (%)	20.7 ± 3.6	14.7 – 27.5

Abbreviations: *ECV*: extracellular volume, *Gd*: gadolinium.

**Figure 2:**
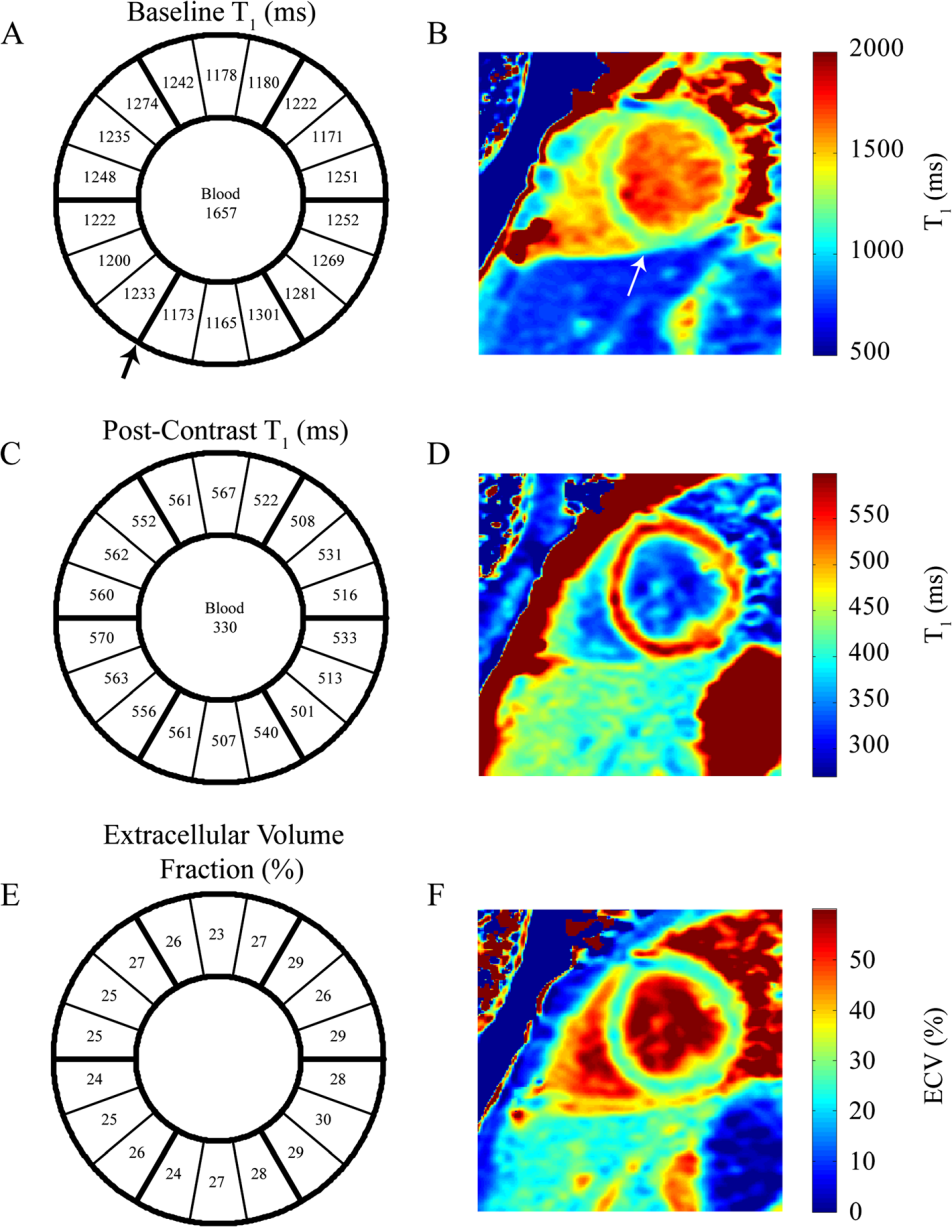
**T**_**1 **_**and ECV mapping analysis in a representative subject. A**) and **B**) show the baseline T_1_ values in the 18 segment model and the corresponding pixel map, respectively. The reference right ventricular insertion point is indicated by the arrow. Similar results for post-contrast T_1_ values are shown in **C**) and **D**), as well as for the calculated extracellular volume fraction, in **E**) and **F**).

A sample T_1_ saturation recovery curve from a single segment as indicated, is shown in Figure 1. Figure 2 shows a representative example of best-fit baseline and post-contrast myocardial and blood T_1_ values, with the 18 segment format shown in A) and C), and with corresponding T_1_ pixel maps in B) and D). Figures 2E) and 2F) show the calculated ECV for this representative example in the18 segment and pixel map formats. Pixel formats are shown here for illustrative purposes. Average baseline T_1_ values (1155.3 ± 56.5 ms) were similar to previous reports using the same method [12,13]. The ECV (20.7 ± 3.6%) was comparable to previous studies in younger adults [14]. Females showed increased non-contrast T_1_ (1191.7 ± 50.0 ms vs 1122.8 ± 37.9 ms) and ECV (23.0 ± 3.7% vs 18.7 ± 3.1%) compared to males.

**Figure 3:**
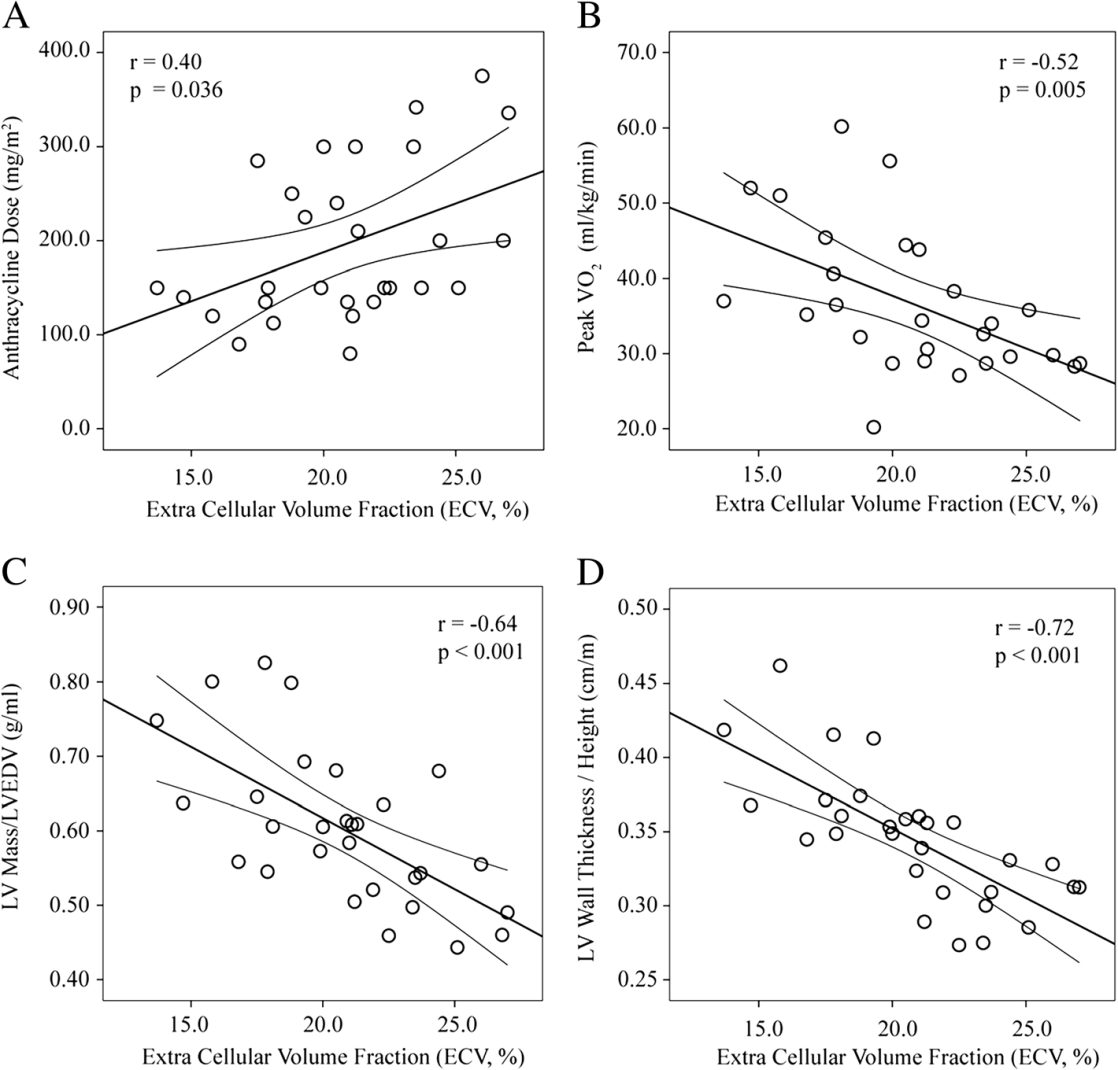
**Correlation of extracellular volume fraction (ECV) with A) anthracycline dose, B) peak VO**_**2**_**, C) left ventricular mass/LVEDV and D) LV wall thickness/height.** Error bars show the 95% confidence interval.

**Figure 4:**
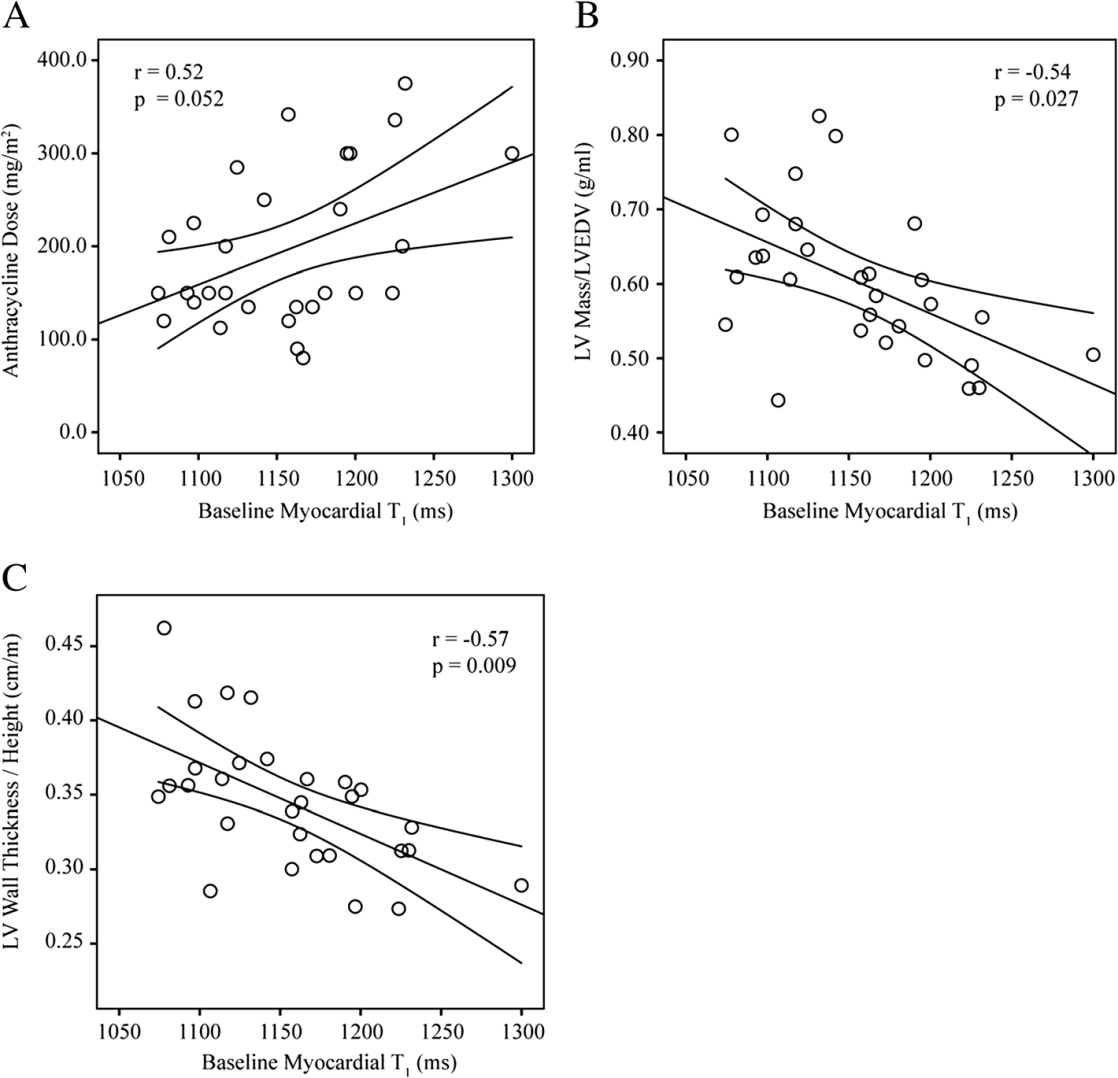
**Correlation of baseline myocardial T**_**1 **_**with A) anthracycline dose and B) left ventricular mass/LVEDV and C) LV wall thickness normalized to height.** Error bars show the 95% confidence interval.

Myocardial ECV showed significant correlations with anthracycline dose (r =0.40, p = 0.036), peak VO_2_ (r = −0.52, p = 0.005), left ventricular mass/volume ratio (r = −0.64, p < 0.001) and wall thickness/height ratio (r = −0.72, p < 0.001) (Figure 3). Baseline myocardial T_1_ values were longer in subjects with larger chemotherapy dose (r =0.52, p = 0.052), reduced mass/volume (r = −0.54, p = 0.027), and reduced wall thickness/height ratios (r = −0.57, p = 0.009), but did not correlate with peak VO_2_ (Figure 4). Myocardial T_2_ values (54.6 ± 4.9 ms) were within normal reported ranges [13,14] and were not significantly correlated with T_1_ or ECV, mass/volume or wall thickness/height ratios. There were no significant relationships between any of the echocardiographic parameters and ECV, T_1,_ or T_2_ values.

### Cardiopulmonary exercise testing

**Figure 5:**
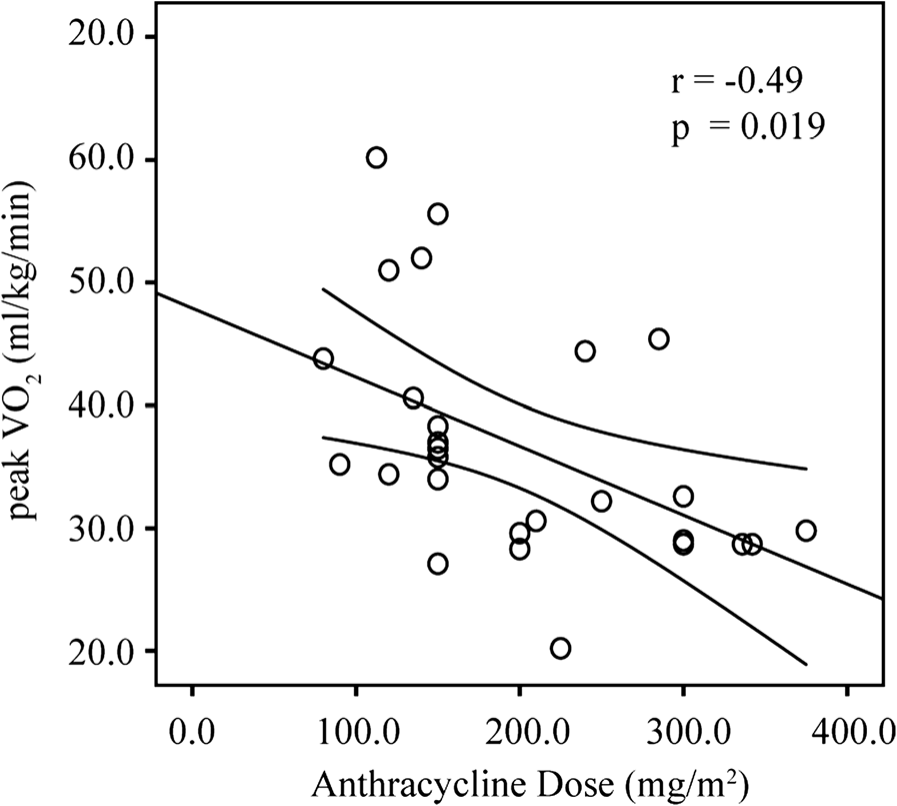
**Correlation of anthracycline dose with peak VO**_**2**_**.** Error bars show the 95% confidence interval.

All but 2 patients underwent exercise testing, due to technical problems with the equipment or being too short for the exercise bike, thus 28 patients were exercised to peak capacity. The mean peak VO_2_ was 35 ± 10 ml/kg/min (83 ± 21% of predicted normal values) and peak HR was 188 ± 17 bpm [15]. There was a significant negative correlation between the anthracycline dose and peak VO_2_ (r = −0.49, p = 0.01, Figure 5).

## Discussion

Our findings show that in asymptomatic survivors of childhood cancer with normal LVEF after anthracycline therapy, peak VO_2_ is reduced and is lower in those who received larger doses of chemotherapy. As a population, parameters of echocardiography tissue deformation were reduced compared to controls, but were still within the normal range and showed no relationship to cumulative dose or exercise capacity. In contrast, the myocardial ECV was significantly related to exercise capacity, anthracycline dose and ventricular remodeling. Subjects with increased ECV had received higher cumulative doses, had reduced exercise capacity and showed early signs of myocardial atrophy including reduced mass/volume and reduced wall thickness. Additionally, baseline myocardial T_1_ values were significantly correlated with mass/volume and wall thickness and had borderline correlations with cumulative dose.

Quantitative myocardial T_1_ mapping, including the derived ECV, are thus potential biomarkers of chemotherapy cardiotoxicity that may have the ability to detect signs of remodeling or tissue damage earlier than conventional functional metrics. These trends, with increased non-contrast T_1_ and ECV with suspected pathology, are consistent with previous reports in hypertrophic and dilated cardiomyopathy [19,20], myocardial infarction [21-23] and several other diseases, however, to our knowledge, no previous T_1_ mapping study has evaluated children. Significantly higher non-contrast myocardial T_1_ and ECV was observed in female subjects in the current study, similar to trends in previous T_1_ mapping studies in older healthy subjects [24,25], and thus may reflect normal gender differences. It is important to note that a wide range of normal myocardial T_1_ and derived ECV values have previously been reported, which reflects systematic differences in the methods. The commonly used MOLLI (modified Look Locker inversion recovery) family of pulse sequences has reported non-contrast myocardial T_1_ values ranging from 939 to 1029 ms at 1.5 T [26-29]. The lower T_1_ values with MOLLI as compared to SASHA are expected and reflect the dependence of the MOLLI method on T_2_ values and inversion pulse efficiency among other factors, including the specific implementation of MOLLI [12,30,31]. A study comparing SASHA and MOLLI yielded significant differences in non-contrast myocardial T_1_ values but similar small ranges of values, 1175 ± 27 ms and 936 ± 25 ms, respectively [12]. These systematic differences in T_1_ values with acquisition method also result in differences in the calculated ECV, and thus care must be taken to interpret these values in the context of the specific acquisition method.

A recent study by Neilan et al. has examined the ECV in adults with previous anthracycline exposure, showing an increased ECV in patients compared to controls [32]. However, their subjects were significantly older than the subjects in the current study (55 years versus 15 years), most subjects displayed overt signs of remodeling, including increased ventricular and atrial volumes, and most were on medications related to heart disease.

Similar to the Neilan study, all patients in our study were negative for late gadolinium enhancement, which is also in agreement with most of the literature to date. Reports of late gadolinium enhancement in late anthracycline cardiotoxicity are few, with subendocardial enhancement being a novel finding and only reported in 2 patients, 10 and 14 years after anthracycline treatment [33]. In contrast, diffuse interstitial myocardial fibrosis is a well-known characteristic of late toxicity following anthracycline therapy [4], and thus there is potential for increased myocardial ECV in these patients, prior to functional abnormalities. Lipshultz et al. observed interstitial fibrosis in myocardial biopsy specimens in conjunction with reduced wall thickness and increased wall stress [34]. Similar pathological findings have been reported in children with doxorubicin-induced congestive heart failure with no evidence of inflammation or necrosis. Goorin et al. showed mild focal interstitial fibrosis 6–10 years after therapy, and Bernaba et al. found interstitial or replacement fibrosis in explanted hearts of adults with anthracycline-induced dilated cardiomyopathy after heart transplantation [35,36]. Importantly, the subjects in the current study received a wide range of anthracycline dose, from 80 to 375 mg/m^2^, and had a wide range of peak VO_2_, ranging from 28 to 60 ml/kg/min, illustrating the broad range of potential chemotherapy toxicity in the study population. The measurement of the correlations of ECV and baseline T_1_ values with the cumulative dose, as well as with morphological changes associated with myocardial atrophy, such as reduced wall thickness normalized to height and reduced mass/volume, was enabled in the current study by the wide range of values for all measures.

CMR measures of global function and mass were largely within the normal range in our patients. Average myocardial T_2_ values in our study were also similar to normal values from previous studies [37,38], indicating no acute inflammation or edema, and with no correlation to therapy dose or exercise capacity. In consideration of factors such as the long interval since chemotherapy, the normal myocardial T_2_ values, and a lack of correlation between T_2_ and T_1_ values in our subjects, it is unlikely that increased baseline T_1_ values observed in the current study are related to edema.

The echocardiographic findings of decreased longitudinal and circumferential strain, and longitudinal SR in patients is comparable with the work of others demonstrating reduced LV myocardial deformation parameters as evidence of subclinical anthracycline cardiotoxicity [7,9,39]. Consistent with the proposition of diastolic dysfunction as an earlier marker of cardiotoxicity [2,9], diastolic abnormalities were observed in our patients, including reduced E’ and A’ velocities, and diastolic SR. Despite the many reports of echocardiographic deformation parameters as potential early markers, their values are often still within the normal range [16], as was found in the current study, indicating that echocardiographic parameters alone may not have sufficient ability to distinguish between normal and abnormal myocardium. Perhaps more importantly, no significant relationship between any echocardiographic functional parameter and anthracycline dose or exercise capacity was observed. Similarly, the echocardiographic functional parameters were not significantly correlated with any CMR-derived parameters. It is possible that the significant association of the CMR tissue characteristics with factors such therapy dose and exercise capacity reflects the closer physical relationship of the tissue status and these factors. In contrast, the functional parameters, particularly measured at rest, are dependent on other confounders such as loading conditions and heart rate which could lower their discriminatory power in terms of underlying tissue damage.

Peak VO_2_ is the gold standard measure of aerobic fitness [40]. De Caro et al. found that reduced peak VO_2_ in patients was able to differentiate between controls and patients post-anthracycline therapy, but that cardiac dysfunction, as measured with echocardiography at rest or with exercise, was not significantly related to reduced exercise capacity. [41]. The significant reduction in peak VO_2_ and its inverse correlation with ECV in our patients suggests a cardiotoxic contribution to the reduced whole body fitness. However, the potential direct toxic effects on skeletal muscle and vascular system, occurring in conjunction with cardiac damage, cannot be excluded and thus the cardiac contribution to reduced peak VO_2_ cannot be determined. Other factors such as respiratory or musculoskeletal function or deconditioning from a sedentary lifestyle following previous chronic illness may also reduce the specificity of peak VO_2_ to cardiac damage [42].

### Limitations

A limitation of the current study is the lack of an age-matched control group for the CMR acquisitions. Nonetheless, the strength of our findings are the significant correlation of ECV with cumulative dose, exercise capacity and structural measures of remodeling, as opposed to their absolute values as compared to controls. Another limitation is the heterogeneous population of diagnoses, radiation, and varying treatment protocols with differing length of follow-up, which were likely important sources of variability for all findings. The study is also limited by the lack of a comparison to a gold stand and measure of fibrosis in subjects with exposure to anthracyclines. Future human or animal studies would ideally compare biopsy evaluation of myocardial microstructure with non-contrast T_1_ and ECV.

## Conclusions

In children, following anthracycline therapy, myocardial T_1_ values and calculated ECV correlate with cumulative chemotherapy dose, exercise capacity and subtle structural remodeling, which may have the potential to characterize myocardial tissue changes prior to functional alterations. These novel measures of diffuse fibrosis may prove to be early non-invasive tissue biomarkers of chronic anthracycline cardiotoxicity in the future.

## Abbreviations

ECV: Extracellular volume fraction; EDV: End-diastolic volume; EF: Ejection fraction; ESV: End-systolic volume; FS: Fractional shortening; IVRT: Isovolumic relaxation time; SR: Strain rate.

## Competing interests

The authors declare that they have no competing interests.

## Authors’ contributions

ET contributed to the conception and design of the study, patient recruitment and consent, and supervised the CMR, echocardiography and exercise testing. MH contributed to the conception and design of the study and performed the exercise testing. KC designed the CMR sequences, performed the CMR and helped to draft the manuscript. MS contributed to the conception and design of the study, and identified suitable patients. SK was involved in patient recruitment, performing the echocardiograms, and analyzed the functional echocardiographic parameters. NK was involved in performing the echocardiograms, and critically revised the manuscript for important intellectual content. JP performed or supervised the CMR and helped to draft the manuscript. AM contributed to the conception and design of the study, provided statistical support, and critically revised the manuscript for important intellectual content. RT designed the CMR sequences, performed or supervised the CMR and drafted the manuscript. All authors read and approved the final manuscript.
